# Landsenses Ecology: A New Idea for Watershed Ecology Restoration

**DOI:** 10.3390/ijerph20043610

**Published:** 2023-02-17

**Authors:** Chang Liu, Jingyi Ouyang, Jinshan Yan, Lina Tang

**Affiliations:** 1 Key Laboratory of Urban Environment and Health, Institute of Urban Environment, Chinese Academy of Sciences, Xiamen 361021, China; 2 University of Chinese Academy of Sciences, Beijing 100049, China

**Keywords:** watershed ecology restoration, landsenses ecology, human perceptions, landsenses ecology restoration method, the indicator system

## Abstract

Watershed ecology restoration is the key to solving the ecological and environmental problems of watersheds and achieving sustainable development. As one direction of the frontiers of ecology, landsenses ecology is supported by science and technology and cares for humans. It has a great significance for enhancing the human habitat and achieving sustainable development. Combining landsenses ecology and the technical process of watershed ecology restoration allows the integration of people’s visions into the system of strategies and applied technologies for watershed ecology restoration while ensuring the restoration of the ecological functions of watersheds. It is a complement to the traditional ecosystem restoration model. This study establishes the connection between landsenses ecology and watershed ecology restoration in terms of goals, models and focus of attention. It aims to construct an indicator system of restoration with the application of landsenses ecology, form a complete process of ecological restoration with the integration of landsenses ecology and apply it to the ecological restoration of watershed elements with relatively intensive human activity such as urban green areas, buildings and wetlands (rivers and lakes). Rather than just always emphasizing natural ecology, landsenses ecology treats human beings as part of nature. It tries to establish a more comprehensive, humanized ideal restoration model by taking “human perceptions” into consideration. Through a restoration process based on long-term and continuous coordination, feedback and improvement, it can improve the ecological benefits of the watershed and improve residents’ well-being, so as to ultimately realize the establishment of a “community of life between man and nature”.

## 1. Introduction

According to the World Population Prospects 2022, the global population is expected to reach 8 billion by the end of 2022 [1]. Feeding the huge number of people puts enormous environmental pressure on our planet [2]. Irrational human industrial activities and urban expansion directly affect landscape patterns and ecological processes, and bring adverse effects such as increased urban CO_2_ concentrations and frequent extreme weather [3,4], posing a direct risk to human health and threatening ecosystem services as well as the sustainable development of human societies [5,6]. At the same time, with the improvement in human living standards, human needs for higher levels of safe, healthy, sustainable and beautiful ecological environments are increasing, and existing urban planning and management should further emphasize the trade-off of multiple interests to achieve coordinated social–economic–natural development. In this new era, how to manage ecological protection and restoration well to achieve the sustainable development of ecosystem services has become a hot issue of common concern for the whole society [7,8].

Ecosystem services refer to the benefits that humans derive from natural systems [9,10]. For watersheds with relatively dense populations, the ecosystem service values are often not equal to the ecological benefits derived from the natural environment [11,12]. The cultural service of ecosystems is the bridge that connects social and ecological elements, which helps to realize sustainable development. It contains aesthetic, spiritual and educational values, and compared to other types of services, it is more likely to directly enhance the well-being of local people [13,14]. However, research on the cultural service of ecosystems is still weak due to its intangible nature and difficulty in terms of its quantification [15].

Watershed ecosystem restoration mainly focuses on the supporting service and the regulating service of the ecosystem, and the current restoration methods focus on the use of engineering and technical means; the selected indicator system and data cannot comprehensively reflect the correlation between environmental changes and human well-being. With the development of big data, GIS, the Environmental Internet of Things and other technologies, the accessibility of indicators about human perceptions, culture and society should be further enhanced in watershed ecosystem restoration practices and a new indicator system for watershed restoration orientated to improve the coordination between ecosystem health and human well-being should be constructed [16,17].

The concept of landsenses ecology provides a powerful complement to the traditional watershed ecology restoration model. Landsenses ecology is a scientific discipline that studies land use planning, construction and management toward sustainable development, based on ecological principles and the analysis framework of natural elements, physical senses, psychological perceptions, socio-economic perspectives, process risk and associated aspects [18]. It treats humans as a part of nature and aims at the realization of harmony between nature and humans, which is in line with the ancient Chinese philosophy of the “unity of man and nature” [19].

Landsenses ecology takes the enhancement of ecosystem services, which are of the most direct benefits to human beings, as its primary task, and landsense creation under its theories is an effective way to strengthen ecosystem services. In order to meet the demand of a “community of life between man and nature”, with landsenses ecology theory as its basis, this paper organically integrates landsense creation with a meliorization model into watershed ecology restoration projects and builds an indicator system of restoration with the application of landsenses ecology, forming a process of watershed ecology restoration based on landsenses ecology. This paper is of positive significance to the concept update, method innovation and practice exploration of watershed ecology restoration.

## 2. Theoretical and Conceptual Framework

### 2.1. The Watershed Ecology Protection and Restoration Practices

The watershed ecology protection and restoration project, based on the overall planning of the national land space and relevant special plans such as ecological protection and restoration of the national land space, refers to the process and activities of the overall protection, systematic restoration and comprehensive management of damaged, degraded and function-declining ecosystems within a certain area aiming to improve the self-recovery capacity and stability of the ecosystems [20].

The technical process of this ecological protection and restoration project is generally divided into four stages: project planning, project design, project implementation as well as project management and maintenance. The project planning stage serves to identify and diagnose macroscopic problems at the regional (or watershed) scale, and formulate protection and restoration objectives. The project design stage mainly serves to diagnose ecological problems of each protection and restoration unit at the ecosystem scale, and formulate corresponding protection and restoration models and measures. The project implementation stage serves the site scale subproject construction. The project management and maintenance, including monitoring assessment and adaptive management, runs through the whole process of the ecological protection and restoration [21].

### 2.2. Landsenses Ecology

#### 2.2.1. The Connotations of Landsenses Ecology

Landsenses ecology is a discipline based on the theories of landscape ecology, environmental psychology and landscape architecture, which studies the interaction between human perceptions and natural and artificial environments through humans’ various senses. It can also be understood as perception ecology. Landsenses ecology emphasizes the collection of people’s perceptions, cognitions and feedback toward landscapes in the planning and design process as well as the synergistic effect of multisensory combination and interaction. It fully considers the matching of the supply of the cultural service of ecosystems, so as to enhance the correlation and harmony among natural landscapes, artificial landscapes and people, and seek the maximum well-being for local people [14,22].

Landsense creation is the main way to put landsenses ecology into practice. By integrating people’s vision into a carrier in some form, a strong connection between people and the carrier can be established. Then through this carrier, people can feel the corresponding vision and their resonance with this vision can be aroused, leading them to have the initiative to protect and maintain such a vision and its carrier. In this way, a tendency to sustainable development is gradually formed, which is also the effect to be pursued in the later stage of landsense creation. The carrier can either be a city, a block or a building, or a painting, poem, song or a logo, etc. The carrier with these kinds of attributes is named as a landsense, and the whole process of the conception and construction of the landsense is considered as landsense creation.

#### 2.2.2. The Way and Main Principles of Landsense Creation

There are three ways for landsense creation. The first is to integrate the vision into the existing carrier to make it a landsense. The second is to reconstruct with certain degrees the existing carrier to form a new carrier according to the needs of presenting the vision, and then integrate the vision into the newly formed carrier to make it a landsense. The third is to directly construct a new carrier and integrate the vision into it according to the needs of presenting the vision, making it a landsense [23].

There are eight principles for landsense creation. The first is the bidirectional presentation of vision, which means that the landsense designer hopes that the vision presented by a landsense can resonate among people and thus become the common vision of them. The second is the vein-compliance in orientation and bearing, which means that a landsense shall be created and designed according to the patterns of waters and mountains, psychological veins (that is, the location and direction of landsense elements are set by following subjective psychological needs) and the needs of the layout of landsense spatial elements. The third is the multiscale (trans-scale or cross-scale) spatiotemporal combination, which means in landsense creation, people’s perceptions and understanding at different times and spaces can be integrated into the same landsense. The fourth is the systematicness of physical senses, which means in landsense creation, people’s physical senses will be studied systematically as a whole. The fifth is the integrity of psychological perceptions, which means in landsense creation, various elements of psychological perceptions shall be studied. The sixth is the interactivity of physical senses and psychological perceptions, which means the connections and mutual influence between the two shall be noticed and emphasized. The seventh is the dissimilarity in culture, which means the differences in traditions, preferences and cultures of different groups should be taken into account in landsense creation. The eighth is the progressive nature of landsense creation, which means landsense creation is a process of continuous improvement rather than a one-off action [23].

## 3. Landsenses Ecology and Watershed Ecology Restoration

### 3.1. The Connection between Landsenses Ecology and Watershed Ecology Restoration

#### 3.1.1. The Goal

Landsenses ecology studies land use planning, construction and management with the goal of enhancing ecosystem services and achieving sustainable development [18], which is consistent with the essential goal of watershed ecology restoration. Both landsenses ecology and watershed ecology restoration attach great importance to the sustainability of the ecological environment and emphasize the harmonious relationship among society, nature and humans to create a good living environment and development space for human beings [24].

#### 3.1.2. The Model

a. The ecological restoration of watersheds in the new era is moving toward holistic protection, systematic restoration and integrated management, which is similar to the holistic view and systematicness emphasized by landsenses ecology.
b. Ecological restoration projects carry out long-term tracking, monitoring and adaptive management, so as to continuously improve the technologies and regulations of ecological protection and restoration projects. Landsenses ecology emphasizes that ecological planning is a process of long-term and continuous coordination, feedback and improvement; that is, the process of meliorization [25,26]. Landsenses ecology uses technologies such as the Internet of Things to acquire dynamic data from multiple pathways, sources and scales to build the meliorization model.
c. There are three common restoration ways for watershed ecology restoration: natural restoration, restoration with assistance and ecological reconstruction [27]. In landsenses ecology, the landsense creation method that incorporates visions into the existing carriers or modifies the existing carriers to form new carriers is similar to the restoration method “restoration with assistance”; another way of landsense creation that constructs new carriers and incorporates visions into them can be achieved by the restoration method “ecological reconstruction”.

#### 3.1.3. The Focus

a. Watershed ecology restoration focuses more on enhancing the supporting and regulating services of the ecosystem. In contrast, landsenses ecology focuses on enhancing the cultural services of ecosystems. It considers fully the regional nature and diversity of cultures, and includes people’s physical senses and psychological perceptions in the category of objects that shall be studied and analyzed, so as to seek the well-being of local people.
b. Watershed ecology restoration focuses more on the improvement of restoration technology and means, while the realization of ecological and sustainable development of watersheds requires not only the support of the hard science as technology but also that of the soft science such as culture and ethics [23]. Landsenses ecology focuses more on the enhancement of cultural services and people’s well-being with regard to regional ecosystems.

### 3.2. The Construction of the Indicator System of Restoration with Application of Landsenses Ecology

The indicator system of restoration with the application of landsenses ecology, which includes the correlation between human perceptions and the cultural service of ecosystems as its starting point, is a powerful complement to the existing ecological restoration indicator systems. Some scholars have constructed multidimensional indicator systems based on the concept of landsenses ecology. Tang et al. (2020) characterized landsenses by two types of indicators: physical senses and psychological perceptions [28]. Zheng et al. (2017) integrated ecological information into an urban planning process on the basis of landsense characteristic indicators, and clarified the challenge and importance of integrating information about residents’ physical senses and psychological perceptions into ecological planning [29].

The construction of the indicator system of restoration with the application of landsenses ecology is based on the existing ecological restoration indicator systems of watersheds. This indicator system takes the improvement in the cultural service of the watershed ecosystem as one of the important tasks of ecological restoration. In the construction of the indicator system, the subjective feelings of the local people are fully considered, and the indicators are constructed from both the aspect of physical senses and the aspect of psychological perceptions. Indicators of physical senses are selected from the following four dimensions [30]: sight, hearing, touch and smell; indicators of psychological perceptions are selected from the three dimensions as follows: psychology, culture and ethics (Table 1).

### 3.3. Process of Watershed Ecology Restoration based on Landsenses Ecology

**Figure 1:**
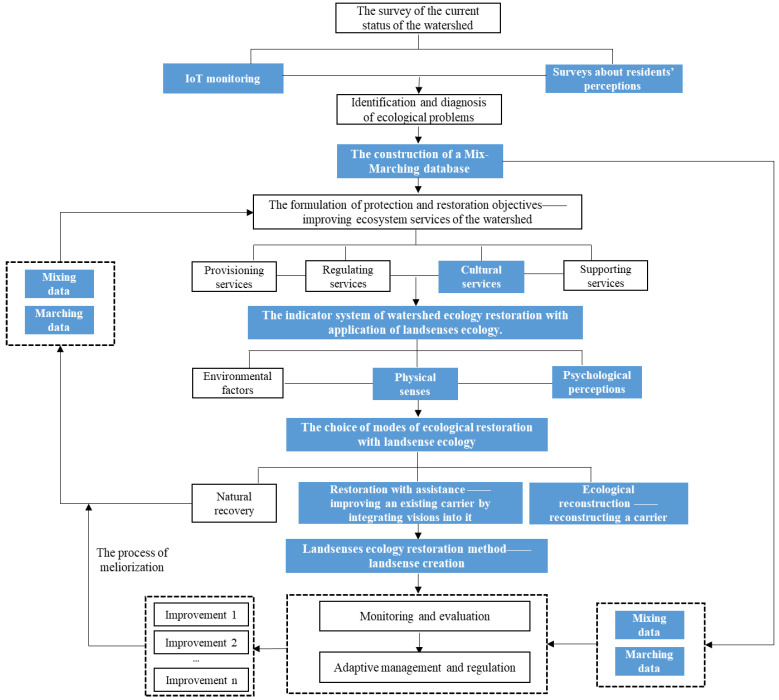
The process of watershed ecology restoration based on landsenses ecology. Note: the blue area is the combination of landsenses ecology and watershed ecology restoration.

This study organically combines the theory of landsenses ecology with the technical process of watershed ecology restoration, and constructs a process of watershed ecology restoration based on landsenses ecology (Figure 1). Compared with traditional ecology restoration, the landsenses ecology restoration method combines IoT monitoring with surveys about residents’ perceptions for data acquisition, which enables more accurate identification and diagnosis of ecological problems in the watershed and is also helpful for the construction of a mix-marching database.

The main task of ecological restoration with landsenses ecology is to enhance the cultural services of the ecosystem. The indicator system of restoration with the application of landsenses ecology is constructed from two aspects: physical senses and psychological perceptions, and the indicators in this system are selected from seven dimensions: sight, hearing, touch, smell, psychology, culture and ethics. With analysis of the ecological conditions and the historical and cultural background of the watershed, suitable carriers will be selected for restoration, and based on the characteristics and current situation of the carriers, a suitable landsenses ecology restoration mode will be chosen. There are mainly two modes in ecological restoration with landsenses ecology: restoration with assistance and ecological reconstruction. Restoration with assistance means to improve an existing carrier by integrating visions into it, while ecological reconstruction means to reconstruct a carrier. The specific restoration plan is made according to the principles of landsense creation. In the restoration process, with the maintenance of the original ecological function of the chosen carrier and by the integrated visions, the carrier is transformed into a landsense, so as to realize the improvement in its cultural services, and ultimately achieve the enhancement of people’s well-being and the maximum benefit of ecology restoration.

The newly constructed landsense can provide mixing data and marching data, i.e., mix-marching data, for the mix-marching database and the later monitoring and evaluation system. This process is helpful for the implementation of adaptive management and regulation work, which enables the achievement of a process of long-term and continuous coordination, feedback and improvement; that is, the process of meliorization.

### 3.4. Application of Landsense Ecology in Watershed Ecology Restoration and its Related Cases

In the project of watershed ecology restoration, landsense creation is carried out on important watershed components such as urban green space, architecture and wetland (river and lake), and with the application of the theories and methods of landsenses ecology, the related landscape patterns can be optimized and the living environment there can also be improved. In the restoration process, as the ecological environment is gradually improved, residents’ ecological and environmental protection awareness is guided and enhanced, which can mobilize their spontaneous ecological protection behaviors and make them participate in the ecological protection project of the watershed. In this way, residents’ visions and social needs are satisfied, which promotes the sustainable development of ecological protection and restoration.

#### 3.4.1. Urban Green Space

As an important component of ecological infrastructure, urban green space is of various ecological and social benefits such as lowering the temperature, increasing the humidity, purifying air, providing biological habitats and recreational areas, etc. Combined with the ecological attributes of the city, such as climatic characteristics and seasonal characteristics, landsense creation is made from the aspects of sight and smell to improve people’s comprehensive sensory experience. In terms of sight, it should be taken into account that plants change with the seasons, and plants’ differences in shapes, colors and styles provide different visual experiences for the visitors. Under the sunlight, the color of plants varies with light and shadow. Making use of the changes in light and shadow can provide visitors with rich visual effects and enrich the spatial layers of the landscape, making the space more rhythmic. In terms of smell, different types of plants emit different fragrances, causing changes in space atmosphere and thus making visitors experience changes psychologically and physically during their visit. Not only do plants emit fragrances, they also produce negative air ions and phytoncides that have potential health benefits for people. Therefore, the proportion of deciduous and evergreen trees should be controlled, and aromatic plants should be selected reasonably, so as to form a woodland landscape with rich species, distinct seasons and a reasonable combination in density from the aspects of space, color and smell [31,32,33].

However, with problems brought about by rapid urban development such as climate change and heat island effect, urban green space as a place for people to play and relax is facing great opportunities and challenges. Thermal comfort in outdoor spaces is of vital importance to human health and well-being, and affects the livability of cities. In a humid–hot region with elevated temperatures, it is difficult to improve the thermal experience of urban residents by changing objective conditions such as the microclimate. From the prospective of landsenses ecology, Liu et al. (2022) created a set of indicators in different dimensions based on the subjective feelings of humans, which included physical sense indicators related to visual, auditory and olfactory perceptions as well as humans’ psychological perception indicators, to explore the influencing factors and mechanisms of a visitor’s thermal comfort in an urban park. They selected physical sense indicators such as spatial coordination, the richness of landscape colors, air freshness, plant smell, sound richness and auditory effect. They found physical senses have a complete and mediating effect on thermal comfort, and the total effect it has on people’s thermal comfort is 70.6%. Specifically, it affects thermal comfort through two mediating variables: thermal sensation and psychological perceptions. Specifically, a 1-unit increase in physical senses produces an increase of 0.518 units in thermal sensation, and an increase of 0.428 units in psychological perceptions. Their findings provide a reference for the ecological restoration of urban park green spaces in the context of global warming [34].

#### 3.4.2. Architecture

The multiscale (trans-scale or cross-scale) spatiotemporal combination in architecture restoration refers to the architectural styles and characteristics developed by modern people on the basis of retaining the relevant traditional styles for adaptation to the new environment, which enables the newly built landsenses to be the embodiment of visions of both the old generations and the new ones. This method allows the innovation of architecture pattern with the combination of the characteristics of the watershed, and also realizes the inheritance of traditional culture and its expression forms, which forms a sustainable development tendency in terms of culture and architecture and avoids the disappearance of traditional elements. It brings reference to the realization of sustainable development from the perspective of modern historical and cultural heritage protection.

According to the principle of the vein-compliance in orientation and bearing in landsenses creation, architecture can present the vein-compliance in orientation and bearing in both a physical and psychological sense. The physical vein-compliance in orientation and bearing shows people’s positive adaptation to the natural environment on the basis of their conformity to the natural objective conditions. The vein-compliance in orientation and bearing in a psychological sense (patterns in vision, or psychological veins) reflects a benign interaction between people’s subjective cognition and the natural environment.

Ouyang et al. (2021) conducted a survey of the architecture pattern of the ancient building complex “The House of Hundred Rooms” located in the East Dragon Village, Jiangxi Province; they found it managed to control its influence on the land around it to a limited scale and level. Instead of making changes in the environment around it, it focused mainly on adjusting itself, and, meanwhile, taking the local topography, climate, culture and customs into consideration, by flexible and creative constructing means, it realized its harmonious relationship with nature—by following the physical vein-compliance in orientation and bearing, this ancient building complex set its outer orientation in a counter-tradition way. Conversely, by following the vein-compliance in orientation and bearing in a psychological sense, it arranged the orientation of its inner rooms in a traditional way. Such an architecture pattern captures the core idea of the ancient Chinese philosophy “unity of man and nature”, and with adaptation to local conditions, it manages to develop a form of “unity of man and nature” of its own characteristics. Meanwhile, it realizes the owner’s artificial setting of the orientation of the inner rooms in this building complex based on his own vision. Such a land use method builds a harmonious coexistence relation between man and nature, which is worthy of learning in the modern era [35].

#### 3.4.3. Wetland (River and Lake)

Wetlands are considered to be one of the most productive ecosystems, providing a variety of ecosystem services such as fishery production, water conservation, water purification, biodiversity maintenance, recreation and ecological education [36,37,38]. While improving the ecological functions of wetland such as soil and water conservation, water purification and biodiversity maintenance, it is necessary to develop its cultural services from the aspects of aesthetics, recreation and ecological education [39,40]. Following the principle of the systematicness of physical senses in landsense creation, the topography and water bodies of wetlands can be sorted out, and the artificial terraces, waterside walkways, viaducts and other ways can be used to create spaces with a diversified atmosphere and scale according to local conditions, so as to enrich the experience of visitors. The viaducts can shorten visitors’ distance from the tree canopy area and enable them to directly touch branches and leaves of the trees, improving visitors’ experience in terms of touch. For the construction of roads and pavements, materials with local features and natural textures should be used to create a spatial atmosphere with wildness and a local cultural style, so as to shorten visitors’ psychological distance from the place. At the same time, ecological exhibition halls and cultural publicity corridors can be constructed to enrich visitors’ psychological cognitive experience. In terms of water body construction, hard ditches can be reconstructed to reshape the shoreline with natural attributes. River shoals with gentle slopes into the water can be created, and there can also be spaces reserved for rivers according to the idea of the dynamic development of rivers, and vegetation filter strips can be built. According to the ebb and flow of the wetlands, the seasonal rain garden and dry stream can be built to combine publicity and education to induce people’s water-enjoying behaviors and enhance their experience and interaction with nature. Taking the application of landsenses ecology in the ecological restoration project of the coastal zone of the Guangdong–Hong Kong–Macao Greater Bay Area as an example, in the Xiangluwan Beach restoration project, for improving the ecological environment of Xiangluwan Beach and its nearby coastal space as well as meeting the recreational needs of residents there, by collecting surveys with information including the status quo of the Bay Area’s natural elements’ and its surrounding landscape elements, etc., taking humanistic, historical and cultural elements of the city as the main reference of the material used in the creation of a landsense carrier, and fully considering the physical perceptions and psychological perceptions of the residents, the researchers managed to make a scientific restoration plan in which the design was integrated with nature on the basis of protecting the original landscape pattern and natural ecosystem. Through the implementation of this plan, the landscape functions of Xiangluwan Beach were transformed, and supporting public facilities such as plant views, beach greenspace and sunshades were built. The whole restoration plan not only met the original needs of restoration, and maintained and improved the recreational service of the area, but also added color to the area from the perspective of aesthetic and psychological feeling enhancement and regional cultural continuity, realizing ecological restoration with added value [27,41].

Wetlands ecology restoration should combine the needs and cultural backgrounds of multi-stakeholders, coordinate and integrate them into a common vision and incorporate them into a carrier. For the cultural services provided by wetlands, based on the principle of the bidirectional presentation of vision in landsense creation, methods such as questionnaire interviews and participatory mapping can be used to identify multi-stakeholder needs for ecosystem services from the perspectives of physical senses and psychological perceptions, map the differences in supply and demand for ecosystem services, and combine planning and design tools to enhance wetland cultural services in a targeted manner [14,23]. At the same time, planners and local government departments should establish wetland management committees for adaptive management and regulation in response to changes in the ecological environment and residents’ needs. The capacity of risk management and emergency planning can also be improved to properly deal with the damage caused by unexpected and special incidents. Taking the application of landsenses ecology in the ecological restoration project of the Muli River in the Guangdong–Hong Kong–Macao Greater Bay Area (GBA) as an example, in planning and implementing, the project took the visual and tactile experience of residents into consideration, enhancing the runoff retention function of the riverbank. Meanwhile, it further improved other functions of the river bank such as cooling and humidifying, and increased the vegetation diversity of it by the construction of a rain garden, and designed the riverbank fence by incorporating the local cultural elements. The project adopted IoT monitoring technology to monitor the microclimate of this river section in real time, so as to achieve adaptive management and regulation [42]. Through surveys of the residents’ satisfaction with the ecological infrastructure constructed and improved during the restoration, and before and after the implementation of the restoration project, Shang et al. (2022) found that the average score of residents’ satisfaction increased by 1.4 points after the restoration, and the proportion of residents with great satisfaction increased from 0.71% to 19.68%. The project managed to improve the well-being of the residents via restoration based on landsenses ecology [42].

Wetland ecology restoration should be carried out with progressive and process-oriented thinking according to the characteristics of wetland ecosystems such as resilience, seasonality and dynamics. According to the principle of the progressive nature of landsense creation, the wetland ecology restoration process can be divided into three main stages: the input stage, the self-sustaining stage and the benefit stage. In the input stage, a landsenses ecology restoration plan is developed based on the data such as regional traditional culture, ecological environment conditions and information about people’s physical senses and psychological perceptions collected from on-site research and IOT monitoring, and a specific plan for landsense creation can also be determined and implemented; in this stage, artificial restoration is the main means. In the self-sustaining stage, the task of landsense creation is basically completed, and the economic benefits generated by the wetland are applied to assist the artificial restoration of the ecosystem. In the benefit stage, the natural restoration state of the ecosystem is achieved by its self-purification ability, forming a complete “landsense ecosystem” that integrates hard with soft science. For example, the watercourse from Houxi river’s low reaches to the Xinglinwan reservoir in Jimei District, Xiamen, needed to remove a large amount of silt during the treatment process in 2010. Now, several islands consciously built up with silt at that time have long been covered with vegetation and have become important habitats for birds, increasing biodiversity and adding life to the riverbank landscape, which reflects the process from the initial investment to the final realization of benefits [27].

## 4. Conclusions

As a complement to the traditional ecosystem restoration model, landsenses ecology focuses on the interaction between the human and natural environment while ensuring the restoration of the regulating and provisioning function of a watershed ecosystem. Through a restoration process including long-term and continuous coordination, feedback and improvement, etc., it achieves the harmonious and sustainable development of the natural environment, society and economy of the watershed. This study establishes the connection between landsenses ecology and ecological restoration in terms of goals, models and focus of attention. Based on a perspective of “human perceptions”, it adds more indicators for watershed ecology restoration, forms a complete process of ecological restoration with the integration of landsenses ecology and applies it to ecological restoration in watershed elements such as urban green areas, buildings and wetlands (rivers and lakes). The results of this study provide a “new idea” for ecological restoration in watersheds with intensive human activities. Instead of only focusing on natural environment, landsenses ecology takes humans as part of nature. It tries to reach the harmony between man and nature to establish a “community of life” between them. This is not only the embodiment of the ancient Chinese philosophy of “tian ren he yi”, which means man is an integral part of nature, but also offers another way of expressing the modern concept of ecological justice.

## Figures and Tables

**Table 1:** The indicator system of watershed ecology restoration with application of landsenses ecology.

	Dimension	Indicators of Landsense	Influence Factor
Physical senses	Visual	Spatial coordination	Plants, roads, buildings and garden sketch
		Visual openness	
		Richness of landscape colors	
	Auditory	Sound richness	Natural sounds, music and noise
		Auditory effect	
	Touch	Temperature sensation	Temperature
		Humidity sensation	Humidity
		Wind speed sensation	Wind
		Sunlight sensation	Light
		Water-touching sensation	Water
		Material texture sensation	Plants, roads, buildings and garden sketch
	Olfactory	Air freshness	Air
		Plant smell	Plant
Psychological perceptions	Psychology	Sense of pleasure	Leisure activities such as sightseeing, exercising or getting close to nature
		Sense of communication	
		Sense of satisfaction	
		Sense of security	
	Culture	Cultural experience	Education and publicity, cultural performances, cultural heritage, buildings
	
	Ethics	Customs and traditions	History and culture, local conditions and customs
		Code of conduct	People’s relationship with people, society and nature
		Moral code	

## Data Availability

Not applicable.
